# Scale-Up, Retention and HIV/STI Prevalence Trends among Female Sex Workers Attending VICITS Clinics in Guatemala

**DOI:** 10.1371/journal.pone.0103455

**Published:** 2014-08-28

**Authors:** Sonia Morales-Miranda, Jerry O. Jacobson, Itzel Loya-Montiel, Ricardo Mendizabal-Burastero, César Galindo-Arandi, Carlos Flores, Sanny Y. Chen

**Affiliations:** 1 Center for Health Studies, Universidad del Valle de Guatemala, Guatemala City, Guatemala; 2 National STI/HIV/AIDS Program, Ministry of Public Health, Guatemala City, Guatemala; 3 Division of Global HIV/AIDS, Centers for Disease Control and Prevention – Central America Regional Office, Guatemala City, Guatemala; Alberta Provincial Laboratory for Public Health/University of Alberta, Canada

## Abstract

**Background:**

Since 2007, Guatemala integrated STI clinical service with an HIV prevention model into four existing public health clinics to prevent HIV infection, known as the VICITS strategy. We present the first assessment of VICITS scale-up, retention, HIV and STI prevalence trends, and risk factors associated with HIV infection among Female Sex Workers (FSW) attending VICITS clinics in Guatemala.

**Methods:**

Demographic, behavioral and clinical data were collected using a standardized form. Data was analyzed by year and health center. HIV and STI prevalence were estimated from routine visits. Retention was estimated as the percent of new users attending VICITS clinics who returned for at least one follow-up visit to any VICITS clinic within 12 months. Separate multivariate logistic regression models were conducted to investigate factors associated with HIV infection and program retention.

**Results:**

During 2007–2011 5,682 FSW visited a VICITS clinic for the first-time. HIV prevalence varied from 0.4% to 5.8%, and chlamydia prevalence from 0% to 14.3%, across sites. Attending the Puerto Barrios clinic, having a current syphilis infection, working primarily on the street, and using the telephone or internet to contact clients were associated with HIV infection. The number of FSW accessing VICITS annually increased from 556 to 2,557 (361%) during the period. In 2011 retention varied across locations from 7.7% to 42.7%. Factors negatively impacting retention included current HIV diagnosis, having practiced sex work in another country, being born in Honduras, and attending Marco Antonio Foundation or Quetzaltenango clinic sites. Systematic time trends did not emerge, however 2008 and 2010 were characterized by reduced retention.

**Conclusions:**

Our data show local differences in HIV prevalence and clinic attendance that can be used to prioritize prevention activities targeting FSW in Guatemala. VICITS achieved rapid scale-up; however, a better understanding of the causes of low return rates is urgently needed.

## Introduction

Guatemala has a concentrated epidemic of human immunodeficiency virus (HIV) with prevalence greater than 5% in at-risk groups, such as men who have sex with men (MSM) and male and female sex workers (MSW, FSW) [1]. Sexual transmission accounts for 93% of the HIV transmission in Guatemala [2]. In 1998, the first study of FSW reported an HIV prevalence of 4.7% (n = 470) in Guatemala City, 11.1% (n = 117) in Puerto Barrios, Izabal (Honduras border), and 2.4% (n = 210) in Escuintla and Puerto San José [3]. In 2003, HIV prevalence was reported at 4.3% in Guatemala City (n = 309) and Puerto Barrios (n = 98) and 2.4% in Escuintla and Puerto San José [4]. In 2006, a study of 300 FSW in Puerto Barrios, Quetzaltenango, Coatepeque, Zacapa, and Guatemala City found an HIV prevalence of 1.1% [5], and in 2010, another study of FSW from 17 departmental areas in the country reported an HIV prevalence of 3.8% [6]. The behavioral surveillance survey from 2012 reported an HIV prevalence of 1.1% (95% CI 0.5–2.4; n = 607) in Guatemala City, 3.7% (95% CI 1.8–6.5; n = 299) in Escuintla/Puerto San José, and 2.0% (CI 95% 0.7–4.3; n = 296) in Malacatán and Tecún Umán (Mexico border) [7].

The potential for sexually transmitted infections (STIs) to facilitate HIV transmission has been widely documented [8]. Data have shown that the control of STI can prevent the acquisition of HIV infection. In 2007, the Guatemala Ministry of Public Health (MSPAS) implemented the Sentinel Surveillance of HIV/STI (VICITS) strategy in existing public health clinics–a strategy that lies within the framework of second-generation HIV surveillance that utilizes STI and behavioral data to inform, prevent, and control the HIV epidemic in key populations in Guatemala. The VICITS clinics serve the general population and key populations such as MSM, FSW, and transgender individuals. They provide more extensive services for the prevention, diagnosis, care, and treatment of STIs to FSW than the general population. These specific services include a comprehensive physical examination with gynecological and anal examinations, laboratory diagnosis of STIs, STI counseling, and access to STI medications and condoms. Currently, there are four VICITS clinics that serve FSW: STI health center in Zone 3 in Guatemala City (since 2007), health center in the city of Puerto Barrios in Izabal bordering Honduras (since 2008), health clinic at Marco Antonio Foundation (FMA), non-governmental organization (NGO) in Guatemala City (since 2010), and a health center in Quetzaltenango, the second largest city in Guatemala (since 2010).

By law, FSW working in Guatemala must register routine visits to health centers in an official booklet to certify that they do not have an STI infection [9]. Since 2007, MSPAS has obtained sentinel surveillance data for HIV, syphilis, chlamydia, and gonorrhea among FSW through the VICITS strategy. In addition to providing sentinel surveillance data for MSPAS program use and action, the VICITS strategy also provides behavioral interventions for the prevention and control of STIs among FSW, such as increasing the knowledge of HIV and STI transmission, encouraging consistent condom use, and distributing condoms at each visit. Specialized counseling for FSW is also provided by health personnel who receive periodic trainings in stigma and discrimination and who have an awareness and understanding of the implication of stigma in creating obstacles to effective HIV prevention.

In the absence of reliable data sources to characterize the HIV and STI epidemic among FSW, the VICITS strategy presents a promising option for lower-middle income countries with limited resources. The VICITS model, which integrates provision of STI diagnostic and treatment services, HIV prevention including condom promotion and behavioral interventions, and routine monitoring of clinical, behavioral, and laboratory data for surveillance purposes, was first implemented in Bolivia [10]. Although on-going collection of HIV and STI sentinel surveillance data among FSW has been conducted in Guatemala since 2007, no analyses have been conducted to explore HIV and STI trends over time or to describe performance in terms of scale-up and retention outcomes.

We analyzed risk behaviors and the prevalence of HIV and STI infection over a 5-year period (2007–2011), and investigated risk factors for HIV infection among FSW attending four VICITS clinics in Guatemala. Additionally, as measures of success of the VICITS strategy, we investigated scale-up, retention, and factors associated with retention at four clinics.

## Methods

### VICITS clinics

We included the four existing VICITS clinics in our analyses: STI health center No. 2 located in zone 3 in Guatemala City (Zone 3); a health center in Puerto Barrios, Izabal (PB); FMA located in zone 4 in Guatemala City; and a health center in Quetzaltenango city.

### FSW VICITS attendees

Women 18 years of age or older who presented for a routine medical visit at one of the four VICITS clinics, who reported exchanging sex for money in the last 12 months, and who agreed to provide consent were recruited into the VICITS program.

### Comprehensive HIV/STI prevention strategy (or VICITS services)

Clinic attendees who met the inclusion criteria received the following services at the first visit: pre-test counseling for HIV/STI including promotion of condom use, physical evaluation (including vaginal and anal exams), HIV testing and etiologic diagnosis of syphilis, genital infection with *Neisseria gonorrhoeae* or *Chlamydia trachomatis*, and syndromic management of STIs, treatment of STIs, as indicated, post-test counseling for HIV, and free condoms. The attending physician or nurse collected data using a standard medical record form from a face-to-face interview on patient demographics, sexual relationships, medical history, and findings from the physical evaluation and laboratory test results at each visit. Demographic information, and alcohol and illicit drug use were assessed during the first visit only. Laboratory test results were assessed every three and six months. After the initial visit, FSW with symptoms or signs of an STI were evaluated at least every 15 days and received presumptive treatment and a reference card for free treatment of their partners at VICITS clinics. FSW who tested positive for HIV were referred to the MSPAS Comprehensive Care Unit for follow up and treatment if indicated.

### Laboratory tests

HIV was diagnosed using rapid tests (Determine) on-site in accordance with the national algorithm for HIV screening and confirmed with Enzyme-Linked Immuno Sorbent Assay (ELISA) at the National Reference Laboratory [11]. Syphilis was diagnosed with reactive Venereal Disease Research Laboratory (VDRL) with *hiso end-point dilution* titer ≥1∶8 and confirmed with treponema rapid test or *Treponema pallidum* particle agglutination (TPPA) at the local laboratory and with ELISA at the National Health Laboratory. Genital infection with *Neisseria gonorrhoeae* or *Chlamydia trachomatis* was diagnosed with PACE2 test (Gen-Probe) at VICITS clinic Zone 3. All STI testing procedures followed the national algorithm [11]. Syphilis and HIV results were delivered 45 minutes after samples were collected. Quality control procedures were performed every three months on 100% of the HIV-positive samples and 10% of the HIV-negative samples at the National Reference Laboratory.

### Statistical analysis

Following each visit, data from the form were entered into an Epi-Info database. The unique patient identifier was an alphanumeric code comprising initials of first and last names, sex, and birth date. In a given year, an active patient was defined as a FSW with at least one registered consultation to a VICITS clinic during the year. The number of FSW attending VICITS clinics each year during 2007–2011 was calculated for each VICITS clinic. We analyzed socio-demographic characteristics (age, marital status, literacy, nationality) and behavioral variables (age at first sexual intercourse, number of years in sex work, number of clients within the past week, consistent condom use with clients in the past 30 days, sex work location (e.g. bar, strip club, brothel or street), tested for HIV in the past 12 months, lifetime drug use, and STI diagnosis in the past 12 months).

Prevalence of HIV and STIs among active FSW in VICITS during 2007–2011 was estimated annually. Prevalence of HIV infection included laboratory results up to and including the respective year. Time trends in STI prevalence were assessed using person-level logistic regression models estimated separately for each VICITS location. The models regressed laboratory-confirmed infection status on year of first visit to identify time trends in prevalence. Transforms of year (square, cubed, square root) were also examined. Time trends in demographic and behavioral characteristics of VICITS clients were assessed similarly. Discrete quantitative variables (e.g. number of partners) were assessed using normal instead of logistic regression models. Separately, person-level logistic regression models controlling for facility assessed factors associated with HIV infection and retention among first-time VICITS clinic attendees. Only known confounders and variables that were statistically significant (p<.10) in univariate analyses were included in the multivariate model. The final model includes adjusted odds ratio significant at the 10% level.

Scale-up of services and retention of clients over time were also assessed as indicators of program performance. Retention was defined as the percentage of patients who returned for one or more follow-up visits to the same or a different clinic within 12 months of the initial visit. Because systematic patterns in retention are relevant to interpreting trends in infection prevalence among active patients, a logistic regression predicting retention was fit to health center, socio demographic characteristics, and behavioral variables. Year of visit was also examined as a covariate in order to estimate trends in retention unexplained by changes in patient characteristics over time. Diagnosis of HIV prior to entering VICITS and during the first VICITS visit was also examined.

### Ethical statements

This is a secondary data analysis, not associated with patient identification data. The protocol was approved by Del Valle University and Centers for Disease Control and Prevention (CDC) Atlanta Ethics Committee.

## Results

### Characteristics of FSW seeking health services at VICITS clinics for the first-time

A total of 5,682 FSW visited one of four VICITS clinics for the first-time from 2007 through 2011. The majority of the FSWs were seen at Zone 3 (Table 1). The median age at all four clinics was 26 years (IQR 22–31), 78.6% were single, 37.9% had less than primary education completed, and 65.9% reported being born in Guatemala. However, at the Puerto Barrios (PB) clinic, which borders Honduras, we observed a higher percentage of FSW of Honduran nationality compared to other clinics. The median number of clients in the past seven days at all clinics was 10 (IQR 5–20), 59.5% reported practicing sex work for less than a year, and 3.6% reported inconsistent condom use with clients in the past 30 days. Approximately 84% reported working primarily at venue-based locations (i.e. strip clubs, brothels based at residential homes or bars) and only 10.8% reported working at street locations. Of note, FSW attending the FMA clinic had different characteristics than other clinics with 29.0% reported being married or cohabitating with a partner, 52.1% having less than primary education completed, 56.7% working primarily on the streets, and 9.6% using condoms inconsistently with clients in the past 30 days (Table 1).

**Table 1:** Sociodemographic and behavioral characteristics of FSW attending VICITS clinics for the first time by clinic, Guatemala, 2007–2011 (N = 5,682).

Clinic	Zone 3	FMA	PuertoBarrios	Quetzaltenango	P value**	Total
	n (%)	n (%)	n (%)	n (%)		n (%)
	N = 3,928	N = 597	N = 575	N = 523		N = 5,623
Year of first visit						
2007	556(14.1)	0	0	0	<.001	556(9.9)
2008	952(24.2)	0	45(7.8	0		997(17.7)
2009	1,119(28.5)	0	186(32.3)	0		1,305(23.2)
2010	748(19.0)	206(34.5)	183(31.8)	10(1.9)		1,147(20.4)
2011	553(14.1)	391(65.5)	161(28.0)	513(98.1)		1,618(28.8)
Median age in years (IQR)	26(22–30)	27(23–34)	25(22–30)	25(22–30)	<.01	26(22–31)
Marital status*					<.001	
Single	2,905(78.6)	412(69.5)	494(85.9)	420(80.3)		4,231(78.6)
Married/cohabiting	500(13.5)	172(29.0)	40(7.0)	53(10.1)		765(14.2)
Separated/Widowed	289(7.8)	9(1.5)	41(7.1)	50(9.6)		389(7.2)
Highest level of educationcompleted*					<.001	
<Primary	1,483(37.7)	311(52.1)	222(38.6)	113(21.6)		2,129(37.9)
Primary	814(20.7)	152(25.5)	165(28.7)	109(20.8)		1,240(22.0)
≥HS	1,631(41.5)	134(22.4)	188(32.7)	301(57.5)		2,254(40.1)
Nationality					<.001	
Guatemala	2,616(66.6)	467(78.2)	258(44.9)	364(69.6)		3,705(65.9)
El Salvador	481(12.2)	88(14.7)	31(5.4)	62(11.8)		662(11.8)
Nicaragua	527(13.4)	19(3.2)	37(6.4)	50(9.6)		633(11.3)
Honduras	289(7.4)	19(3.2)	247(43.0)	42(8.0)		597(10.6)
Other	15(0.4)	4(0.7)	2(0.3)	5(1.0)		26(0.5)
Pregnant					<.001	
Median age at first sexualintercourse (IQR)	16(15–17)	15(14–17)	15(14–17)	16(15–17)		16(14–17)
Age at first sexualintercourse					<.001	
<15	883(23.6)	197(33.0)	180(31.4)	110(21.0)		1,370(25.2)
15–17	2,076(55.5)	289(48.4)	303(52.8)	301(57.5)		2,969(54.6)
≥18	782(20.9)	111(18.6)	91(15.8)	112(21.4)		1,096(20.2)
Time in sex work*					<.001	
≤1 year	2,295(64.6)	128(33.2)	359(63.1)	212(40.6)		2,994(59.5)
>1 year	1,257(35.4)	257(66.7)	210(36.9)	310(59.4)		2,034(40.4)
Ever practiced sex workin another country	206(6.0)	17(4.3)	75(13.6)	41(7.8)	<.001	339(6.9)
Median number of clientsin the past 7 days	10(5–24)	15(7–25)	4(2–7)	15(10–20)		10(5–20)
Consistent condom usewith clients in the past30 days*	3,650(97.0)	524(91.4)	533(95.5)	509(98.4)	<.001	5,216(96.4)
Sex work location*					<.001	
Strip club	1,693(43.1)	8(1.3)	59(10.3)	339(64.8)		2,099(37.3)
Bar	1,051(26.8)	83(13.9)	480(83.5)	124(23.7)		1,738(30.9)
Street	263(6.7)	338(56.7)	2(0.3)	6(1.1)		609(10.8)
Brothels based at residential homes	791(20.1)	14(2.3)	24(4.2)	39(7.5)		868(15.4)
Other	130(3.3)	153(25.7)	10(1.7)	15(2.9)		308(5.5)
Tested for HIV in thelast 12 months	1,075(38.4)	458(77.0)	551(95.8)	474(90.6)	<.001	2,558(57.0)
Know HIV positivestatus in the first visit	4(0.4)	7(1.5)	14(2.0)	1(0.2)	<.001	26(1.0)
Ever used drugs	922(26.0)	68(11.5)	154(26.8)	17(3.2)	<.001	1,161(22.2)
STI diagnosis in thepast 12 months	420(10.7)	55(9.2)	28(4.9)	87(6.6)	<.001	590(10.5)

*Totals do not add up due to missing observations.

**Chi-square test for categorical variables and Mann-Whitney-Wilcoxon test for continuous variables.

### Prevalence of HIV and other STI

Annual HIV prevalence at each of the clinics is shown in Table 2. HIV prevalence was greatest among FSW seen at the PB site, with no statistical difference over the years. *Chlamydia* had the highest prevalence of all other STIs at all four clinics. The annual chlamydia and syphilis prevalence in Zone 3 and PB remained constant over time while the prevalence of gonorrhea increased in PB (0% in 2009, 0% in 2010, 4.4% in 2011; p = .04) and remained constant in Zone 3.

**Table 2:** Prevalence of HIV and other STI among FSW attending VICITS clinics for the first-time by clinic, Guatemala, 2007–2011.

Clinic	2007		2008		2009		2010		2011		
	n/N	%	n/N	%	n/N	%	n/N	%	n/N	%	P value
Zone 3											
Chlamydia	6/168	3.6	20/414	4.8	9/138	6.5	13/221	5.9	0/43	0.0	0.74
Gonorrhea	0/168	0.0	6/414	1.4	2/138	1.4	0/221	0.0	0/43	0.0	0.46
Syphilis	8/507	1.6	10/698	1.4	5/692	0.7	3/546	0.5	5/464	1.1	0.15
HIV	5/509	1.0	10/715	1.4	6/703	0.9	2/547	0.4	7/462	1.5	0.85
FMA											
Syphilis	-	-	-	-	-	-	8/185	4.3	6/287	2.1	0.19
HIV	-	-	-	-	-	-	3/187	1.6	9/382	2.4	0.56
Puerto Barrios											
Chlamydia	-	-	-	-	4/28	14.3	8/98	8.2	3/45	6.7	0.27
Gonorrhea	-	-	-	-	0/28	0.0	0/98	0.0	2/45	4.4	0.04
Syphilis	-	-	0/5	-	2/70	2.9	5/118	4.2	1/104	1.0	0.46
HIV	-	-	0/5	-	4/86	4.7	4/117	3.4	6/102	5.8	0.56
Quetzaltenango											
HIV	-	-	-	-	-	-	0/2	-	2/259	0.8	-

Notes: HIV measure takes into account tests prior to and including the respective year; other STI measures include results during the year. Results not shown for n<25. Chlamydia and gonorrhea results not available at Quetzaltenango and FMA clinics. Syphilis results not available at the Quetzaltenango clinic.

In multivariate analysis, risk factors for HIV infection during 2007–2011 included attending the PB clinic (adjusted odds ratio [AOR] 4.52, 95% CI 2.09–9.78), having less than primary education vs. primary education (AOR 1.96, 95% CI 1.08–3.57), current syphilis infection (AOR 4.32, 95% CI 1.46–12.81), working primarily on the street (AOR 4.14, 95% CI 1.60–10.69), and using the telephone or internet to contact clients (AOR 3.40, 95% CI 1.11–10.38) (Table 3).

**Table 3:** Risk factors associated with HIV infection among FSW attending VICITS clinics for the first time, Guatemala, 2007–2011 (N = 4,021).

Variable	OR (95% CI)	AOR (95% CI)
Age		
18–24	1.00	
25–34	1.09 (0.61–1.95)	
≥35	2.10 (1.04–4.24)	
Clinic		
Zone 3	1.00	1.00
FMA	2.09 (1.06–4.10)	0.73 (0.31–1.71)
Puerto Barrios	4.58 (2.40–8.74)	4.52 (2.09–9.78)
Quetzaltenango	0.75 (0.18–3.15)	1.11 (0.26–1.71)
Highest level of education completed		
<Primary	1.00	1.00
Primary	0.44 (0.24–0.81)	0.51 (0.28–0.93)
≥HS	0.54 (0.26–1.11)	0.73 (0.35–1.54)
Current syphilis infection	6.13 (2.13–17.59)	4.32 (1.46–12.81)
Sex work location		
Strip club	1.00	1.00
Bar	2.42 (1.17–5.02)	1.37 (0.61–3.11)
Street	3.69 (1.67–8.18)	4.14 (1.60–10.69)
Brothels based at residential homes	0.94 (0.33–2.72)	1.01 (0.35–2.94)
Other (telephone or internet)	3.13 (1.15–8.54)	3.40 (1.11–10.38)

### Scale-up

**Figure 1:**
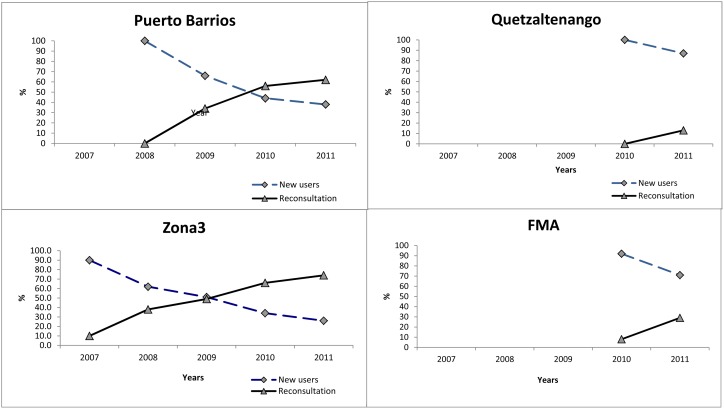
Number of active FSW attending VICITS clinics over time by clinic, Guatemala, 2007–2011.

The four project sites initially experienced significant growth in the number of active (first-time and returning) FSW clients (Figure 1). At the Zone 3 site, the number of FSW increased at an annual average of 85% from 2007 to 2009 and subsequently decreased at 8% annually during 2010 and 2011. At the PB site (on the Caribbean coast bordering Honduras), VICITS scaled up from 356% since the first year and by 19% annually thereafter.

### Retention

Overall, retention (FSW returning to the same or different clinic within 12 months) was low across all four sites during 2007 through 2011. Quetzaltenango had the lowest retention rate in 2011, followed by FMA and PB. Our data show an increase in retention from 39.4% in 2007 to 42.7% in 2011 at the Zone 3 site and a decrease from 48.9% in 2008 to 37.3% in 2011 at PB (Table 4). In a multivariate logistic regression model controlling for age and education, factors associated with negatively impacting retention included having a current HIV diagnosis, ever practicing sex work in another country, being born in Honduras, presenting for the first consultation to VICITS at the FMA or Quetzaltenango clinic sites, and visiting a VICITS clinic for the first-time in 2008 or 2010 (Table 5). Systematic time trends with respect to retention were not detected (Table 5).

**Table 4:** Distribution of FSW who returned for a follow-up visit within 1 year of initial visit by clinic, Guatemala, 2007–2011.

Clinic	2007	2008	2009	2010	2011
	% (n)	% (n)	% (n)	% (n)	% (n)
Zone 3	39.4 (554)	33.1 (949)	40.5 (1136)	34.5 (751)	42.7 (368)
Puerto Barrios	-	48.9 (45)	40.9 (186)	32.8 (183)	37.3 (110)
FMA	-	-	-	11.7 (206)	33.5 (275)
Quetzaltenango	-	-	-	0.0 (10)	7.7 (377)

Note: Data include patients with initial visits up to August 20, 2011.

**Table 5:** Factors associated with retention among FSW attending VICITS clinics for the first-time, Guatemala, 2007–2011 (N = 4,449).

Variable	OR (95% CI)	AOR (95% CI)
Facility		
Zone 3	1.00	1.00
FMA	0.53 (0.43–0.66)	0.38 (0.27–0.52)
Quetzaltenango	0.14 (0.09–0.20)	0.11 (0.07–0.17)
Puerto Barrios	1.02 (0.85–1.23)	1.06 (0.85–1.32)
Year of first visit		
2007	1.00	1.00
2008	0.79 (0.63–0.98)	0.77 (0.62–0.97)
2009	1.05 (0.86–1.29)	1.01 (0.82–1.25)
2010	0.66 (0.53–0.81)	0.76 (0.60–0.95)
2011	0.61 (0.49–0.75)	1.12 (0.87–1.45)
Age	1.09 (0.96–1.23)	1.01 (1.00–1.02)
Highest level of education completed		
<Primary	1.00	1.00
Primary	1.03 (0.91–1.17)	1.02 (0.88–1.18)
≥HS	0.79 (0.67–0.93)	0.85 (0.71–1.02)
Nationality		
Guatemala	1.00	1.00
El Salvador	1.02 (0.85–1.23)	1.00 (0.82–1.24)
Nicaragua	1.08 (0.90–1.29)	1.05 (0.86–1.28)
Honduras	0.84 (0.69–1.02)	0.77 (0.61–0.97)
Other	0.48 (0.18–1.28)	0.50 (0.17–1.53)
Ever practiced sex work in another country	0.77 (0.60–0.99)	0.75 (0.57–0.98)
Current HIV diagnosis	0.43 (0.21–0.90)	0.39 (0.18–0.85)
Ever used drugs	1.14 (0.99–1.31)	
Sex work location		
Strip club	1.00	
Bar	1.09 (0.95–1.26)	
Street	1.06 (0.86–1.31)	
Brothels based at residential homes	1.13 (0.95–1.34)	
Other*	0.68 (0.52–0.90)	

*Telephone or internet contacts.

## Discussion

Our data show local differences in HIV prevalence, patients characteristics, and clinic attendance that can be used to prioritize prevention activities targeting FSW in Guatemala. We found a low prevalence of HIV and other STIs among FSW attending VICITS clinics during 2007–2011. This trend was consistent with the findings from the behavioral surveillance survey implemented in 2012, which reported an HIV prevalence of 1.1% (95% CI. 5%–2.4%) in Guatemala City [7].

Also, we found no statistically significant differences in the prevalence of chlamydia, gonorrhea, syphilis, and HIV over time at the longest running VICITS clinic (Zone 3). These findings contrast with the report from the UALE project, another sentinel surveillance project implemented in Guatemala. They found a significant decline in gonorrhea, chlamydia, trichomoniasis, and candidiasis in follow-up visit during a 3.5 year period in Escuintla Guatemala. However, this finding could be attributed to the differences in demographic characteristics of FSW enrolled in the UALE project (e.g., higher illiterate rate among FSW in UALE compared to FSW in Zone 3) [12]. The PB site, which borders Honduras, reported the highest proportion of immigrant FSW. This group of FSW showed the highest HIV and chlamydia prevalence across VICITS clinics, but lower than the data reported by a similar sentinel strategy in Honduras [13]. The correlation of border and high STI prevalence was also described in another study conducted near the border of Guatemala and Mexico [14].

We also found that the VICITS surveillance and prevention strategy achieved rapid scale-up during 2007–2011. Although all four sites experienced a substantial increase in the number of FSW attending VICITS clinics for the first-time after the first year of implementation, we have observed a gradual decrease in the number of new VICITS clinic attendees at the longest running clinic in Guatemala City (Zone 3) since 2010. The opening of a second VICITS clinic in Guatemala City in 2010 can partially explain for this decrease at Zone 3, as both clinics serve FSW in Guatemala City. These findings suggest that the VICITS strategy is feasible and well accepted, at least initially, by FSW.

Low retention figures were found is all sites, but Quetzaltenango showed lowest retention rate across the four clinics. These results might be influenced by the mobility of the population and the lack of data from regular STI services in the country. However, these finding were similar to the data from Honduras that showed nearly half of FSW returning to follow up visits [13]. As we explore other methods to increase retention at VICITS sites, the experience and data from Peru suggests that the use of mobile clinics might increase the reach and retention of FSW at VICITS clinics [15].

Results from multivariate logistic regression showed that FSW who were diagnosed with an HIV infection at first-visit were less likely to return to the same or different VICITS clinic (retention) for re-testing and follow up. In Guatemala, national guidelines for HIV diagnosis makes obligatory reference to clinical care units for HIV follow up, but common illness, such as STIs, should continue to be treated at secondary care level (where VICITS clinics are). The loss of follow up of these patients might be due to this duality, but it might be also explained by dissatisfaction of public health services, as reported by other studies in Guatemala [16]. The VICITS strategy needs to focus in strengthening close follow up in order to improve retention, or in improving newer methodologies to reach and retain FSW. A better understanding of the causes of low return rates by first-time users at all clinics are urgently needed to enhance accuracy of sentinel surveillance data collected and to improve VICITS program retention.

Our findings are subject to several limitations. First, FSW attending VICITS clinics may not be representative of the FSW population in Guatemala. However, these estimates only reflect FSW attending VICITS clinics located in Guatemala’s largest cities. Second, only FSW seeking health services at VICITS clinics are included in the analysis. Although all FSW must register routine visits in an official booklet, they can be seen at any health center of the MSPAS. Individuals who promote the VICITS strategy coordinate outreach activities with local NGOs, conduct weekly visits to places where FSW solicit business, and monitor social networking sites that promote the VICITS strategy on a weekly basis. They contact FSW and encourage them to seek health services at VICITS sites to increase testing coverage of the FSW population. Third, data are self-reported and social desirability may underestimate the reporting of stigmatized behaviors [11]. Counselors and clinical staff at VICITS clinics are trained to reduce stigma and discrimination towards key populations. Fourth, we did not collect information on sex behavior with type of partner, which might explain the dynamics of HIV and STI transmission in Guatemala.

Despite these limitations, we contend that our VICITS strategy increases access to HIV and STI testing, thus increasing the possibility of early diagnosis of HIV-positive FSW and linking them successfully to care and treatment. In the current context of HIV surveillance and prevention in Guatemala, the VICITS strategy provides critical preventive and care services to key populations, while simultaneously monitoring the epidemic through the systematic collection of routine and programmatic data at existing public health clinics, without the need to conduct separate surveillance activities over time. This strategy provides timely surveillance data and important behavioral information for the design of new prevention activities that can strengthen existing prevention strategies to reduce HIV transmission in Guatemala.
